# Subclassification of B-acute lymphoblastic leukemia according to age, immunophenotype and microenvironment, predicts MRD risk in Mexican children from vulnerable regions

**DOI:** 10.3389/fonc.2023.1304662

**Published:** 2024-01-05

**Authors:** Rubí Romo-Rodríguez, Gabriela Zamora-Herrera, Jebea A. López-Blanco, Lucero López-García, Arely Rosas-Cruz, Laura Alfaro-Hernández, César Omar Trejo-Pichardo, Dulce Rosario Alberto-Aguilar, Diana Casique-Aguirre, Armando Vilchis-Ordoñez, Juan Carlos Solis-Poblano, Lilia Adela García-Stivalet, Vanessa Terán-Cerqueda, Nuria Citlalli Luna-Silva, Miguel Ángel Garrido-Hernández, Lena Sarahí Cano-Cuapio, Karen Ayala-Contreras, Fabiola Domínguez, María de los Ángeles del Campo-Martínez, Gerardo Juárez-Avendaño, Juan Carlos Balandrán, Sonia Mayra Pérez-Tapia, Carlos Fernández-Giménez, Pedro A. Zárate-Rodríguez, Enrique López-Aguilar, Aurora Treviño-García, Célida Duque-Molina, Laura C. Bonifaz, Juan Carlos Núñez-Enríquez, Mariana Cárdenas-González, Elena R. Álvarez-Buylla, Dalia Ramírez-Ramírez, Rosana Pelayo

**Affiliations:** ^1^ Laboratorio de Citómica del Cáncer Infantil, Centro de Investigación Biomédica de Oriente, Instituto Mexicano del Seguro Social, Puebla, Mexico; ^2^ Instituto de Fisiología, Benemérita Universidad Autónoma de Puebla, Puebla, Mexico; ^3^ Facultad de Ciencias Químicas, Benemérita Universidad Autónoma de Puebla, Puebla, Mexico; ^4^ Consejo Nacional de Humanidades, Ciencias y Tecnologías (CONAHCYT), Mexico City, Mexico; ^5^ Hospital Infantil de México ‘Federico Gómez’, Secretaría de Salud, Mexico City, Mexico; ^6^ Servicio de Hematología, Unidad Médica de Alta Especialidad, Hospital de Especialidades “Manuel Avila Camacho”, Instituto Mexicano del Seguro Social, Puebla, Mexico; ^7^ Hospital de la Niñez Oaxaqueña, Secretaría de Salud, Oaxaca, Mexico; ^8^ Hospital del Niño Poblano, Secretaría de Salud, Puebla, Mexico; ^9^ Hospital Infantil de Tlaxcala, Secretaría de Salud, Tlaxcala, Mexico; ^10^ Centro de Investigación Biomédica de Oriente, Instituto Mexicano del Seguro Social, Puebla, Mexico; ^11^ Coordinación de Atención Oncológica, Instituto Mexicano del Seguro Social, Mexico City, Mexico; ^12^ Laboratorio Juárez, Oaxaca, Mexico; ^13^ Department of Pathology, New York University (NYU) School of Medicine, New York, NY, United States; ^14^ Unidad de Desarrollo e Investigación en Bioterapéuticos (UDIBI), Escuela Nacional de Ciencias Biológicas, Instituto Politécnico Nacional, Mexico City, Mexico; ^15^ Cancer Research Center-Instituto de Biología Molecular y Celular del Cáncer-Universidad de Salamanca-Centro Superior de Investigaciones Científicas (IBMCC-USAL-CSIC), Department of Medicine and Cytometry Service-Nucleus Platform, Centro de Investigación Biomédica en Red de Cáncer (CIBERONC), Biomedical Research Institute of Salamanca (IBSAL), University of Salamanca, Salamanca, Spain; ^16^ Hospital Central Sur de Alta Especialidad Pemex, Mexico City, Mexico; ^17^ Organo de Operación Administrativa Desconcentrada, Instituto Mexicano del Seguro Social, Puebla, Mexico; ^18^ Dirección de Prestaciones Médicas, Instituto Mexicano del Seguro Social, Mexico City, Mexico; ^19^ Coordinación de Investigación en Salud, Instituto Mexicano del Seguro Social, Mexico City, Mexico; ^20^ Unidad Médica de Alta Especialidad (UMAE) Hospital de Pediatría “Dr. Silvestre Frenk Freund” Centro Médico Nacional Siglo XXI, Instituto Mexicano del Seguro Social, Mexico City, Mexico; ^21^ Unidad de Educación e Investigación, Instituto Mexicano del Seguro Social, Mexico City, Mexico

**Keywords:** tumor microenvironment, risk stratification, ProB-ALL, measurable residual disease (MRD), Mexican children, acute leukemia

## Abstract

**Introduction:**

The decisive key to disease-free survival in B-cell precursor acute lymphoblastic leukemia in children, is the combination of diagnostic timeliness and treatment efficacy, guided by accurate patient risk stratification. Implementation of standardized and high-precision diagnostic/prognostic systems is particularly important in the most marginalized geographic areas in Mexico, where high numbers of the pediatric population resides and the highest relapse and early death rates due to acute leukemias are recorded even in those cases diagnosed as standard risk.

**Methods:**

By using a multidimensional and integrated analysis of the immunophenotype of leukemic cells, the immunological context and the tumor microenvironment, this study aim to capture the snapshot of acute leukemia at disease debut of a cohort of Mexican children from vulnerable regions in Puebla, Oaxaca and Tlaxcala and its potential use in risk stratification.

**Results and discussion:**

Our findings highlight the existence of a distinct profile of ProB-ALL in children older than 10 years, which is associated with a six-fold increase in the risk of developing measurable residual disease (MRD). Along with the absence of CD34^+^ seminal cells for normal hematopoiesis, this ProB-ALL subtype exhibited several characteristics related to poor prognosis, including the high expression level of myeloid lineage markers such as MPO and CD33, as well as upregulation of CD19, CD34, CD24, CD20 and nuTdT. In contrast, it showed a trend towards decreased expression of CD9, CD81, CD123, CD13, CD15 and CD21. Of note, the mesenchymal stromal cell compartment constituting their leukemic niche in the bone marrow, displayed characteristics of potential suppressive microenvironment, such as the expression of Gal9 and IDO1, and the absence of the chemokine CXCL11. Accordingly, adaptive immunity components were poorly represented. Taken together, our results suggest, for the first time, that a biologically distinct subtype of ProB-ALL emerges in vulnerable adolescents, with a high risk of developing MRD. Rigorous research on potential enhancing factors, environmental or lifestyle, is crucial for its detection and prevention. The use of the reported profile for early risk stratification is suggested.

## 1 Introduction

In Mexico, acute leukemias (AL) stand as the primary cause of mortality due to disease in children. Over a 20-year period (1998-2018), there has been a significant rise in mortality rates from AL in individuals younger than 19 years of age (1, 2). AL account for more than 50% of childhood cancer cases, with an overall survival rate close to 50%, and the age group 0-4 showing the highest incidence rate, while the group aged 15-19 experiencing the highest aggressiveness and mortality rates (3, 4). AL are characterized by the uncontrolled proliferation of oligoclonal precursors, either lymphoid or myeloid, within the bone marrow (BM), where pathological hematopoiesis coexists with residual hematopoiesis, leading to dysfunction in the formation of all types of blood cells due to tumor growth (5, 6).

Of note, it has recently been suggested that the genetic landscape of Mexicans may influence the biology and manifestation of leukemia, and contribute to the increase in the number of people with high-risk ALL (7, 8). Furthermore, the impact of social inequality on childhood cancer mortality rates in Mexico cannot be ignored, since disproportionately higher mortality rates are registered in States characterized by high or very high levels of marginalization (3). Moreover, clear differences in the incidence and risk prognosis of ALL are shown among age groups, with male adolescents being target of poor prognosis diseases (2). Such heterogeneity is not only associated with the molecular identity of the malignant tumor but is also a reflection of the social, economic, and geographical diversity of the Country, which denotes substantive gaps in access to health services, socioeconomic status, and environmental exposition. Indeed, the States of Puebla and Oaxaca, where marginated populations have limited access to health services and socioeconomic or environmental vulnerability are prevalent, have shown constant mortality increase (4). Thus, regionalization of childhood leukemia requires a systemic approach allowing the evaluation of environmental factors to establish more precise clinical and research approaches for the development of therapies adapted to local populations. Likewise, the identification of local risk factors involved in the development of leukemia is crucial for the correct sub-stratification of patients, registry, and prevention.

An early and accurate diagnosis to identify and classify leukemic cells is essential for appropriate risk stratification and the establishment of personalized treatment plans, less intensive and toxic for standard-risk cases compared to high-risk diagnosis. Unfortunately, only approximately 17% of cases at diagnosis exhibit genetic rearrangements associated with prognosis, which limits risk stratification. Furthermore, relapse frequency in Mexican standard-risk patients is 55%, suggesting that the sub-stratification of these patients is crucial (1–4, 6).

Immunophenotyping by multiparametric flow cytometry plays a pivotal role in the precise characterization and quantification of AL burden, making it invaluable for accurate classification of prognostic subgroups and monitoring treatment responses through the detection of MRD. It allows the classification into acute lymphoid leukemia (ALL), which can be further categorized as B-ALL or T-ALL, acute myeloid leukemia (AML) and mixed phenotype acute leukemia (MPAL), depending on the lineage precursor involved. Furthermore, several genetic features of leukemic cells are associated with the expression of specific antigens, aiding in risk group stratification (9). Given the high heterogeneity of the disease, it is crucial to identify immunophenotypic profiles that facilitate the sub-classification and risk stratification to contribute to the ongoing efforts to reduce mortality rates in vulnerable populations. At least three different clusters, within B-ALL, depending on the differentiation stage of the leukemic blasts have been clearly identified in Mexican children: ProB (CD34^+^CD10^+^CD19^+^), PreB (CD34^-^CD10^+^CD19^+^) or the combination of both ProB and PreB precursors (10).Moreover, hematopoietic stem cells (HSCs) reside and are maintained in specialized microenvironments within the BM known as niches, which are comprised of various cell types, including stromal cells, particularly mesenchymal stromal cells (MSCs) (11). BM niches play an important role in sustaining hematopoiesis (12), by influencing different functions of HSCs such as homing, mobilization, quiescence, self-renewal or lineage commitment, protecting the HSCs pool integrity, maintaining immune privileged zones to safeguard HSCs against insults or attack by immune cells (13). In ALL, leukemic cells hijack BM niches, promoting leukemia expansion and creating sanctuaries for tumor cells, in which MSCs play a fundamental role in the pathogenesis and drug resistance of B-ALL cells during chemotherapy, allowing for the reemergence of the disease (14–16). The evaluation of niches, particularly MSCs, offers new therapeutic opportunities due to their negative effect, which corresponds to a critical issue in the treatment of cancer patients.

Given the relevance of urgently addressing the early mortality of Mexican children with leukemia from vulnerable geographic regions in the Country, in this work, we have focused the subclassification by immunophenotype on the identification of risk profiles, considering, not only the tumor characteristics, but the immunological and microenvironmental context of the patients. Our results suggest the occurrence of a biologically distinct subtype of ProB-ALL in adolescents older than 10 years, with associated risk of MRD and a potentially suppressive niche.

## 2 Methods

### 2.1 Patient characteristics and sample collection

This research was performed in accordance with the Declaration of Helsinki and was approved by the Ethics, Research and Biosafety Committees from the National Committee of Scientific Research at IMSS (R-2020-785-177). All samples were collected after informed consent from parents. The study included 159 pediatric patients diagnosed with acute leukemia, who were referred to the Oncoimmunology and Cytomics Laboratory at IMSS for immunophenotyping test. This cohort consisted of pediatric patients from March 2022 to June 2023 attended at the Unidad Médica de Alta Especialidad (UMAE) Hospital de Especialidades IMSS, Hospital para el Niño Poblano, Hospital Infantil de Tlaxcala, Hospital de la Niñez Oaxaqueña and Hospital General de Zona No.1 IMSS. Control BM (No-leukemic patients) were obtained from patients with suspected leukemia but after immunophenotyping no blast cells were detected in the BM sample. BM specimens were collected by aspiration before any treatment and according to international and institutional guidelines.

### 2.2 Immunophenotyping and classification of acute leukemia

BM samples were stained and acquired for flow cytometry analysis according to EuroFlow guidelines. First, samples were stained using the Acute Leukemia Orientation Tube (ALOT) to determine the lineage of immature blast cell populations. We classified AL in five categories according to the affected cell lineage: ProB-ALL (CD34^+^ CD19^+^ cyCD79a^+^), ProB-PreB-ALL (CD34^-/+^ CD19^+^ cyCD79a^+^), PreB-ALL (CD34^-^ CD19^+^ cyCD79a^+^), T-ALL (cyCD3^+^ smCD3^lo^ CD7^+^) and AML (cyMPO^+^ or CD7^+^cyCD3^-^). Once identified the malignant hematopoietic lineage, complementary antibody panels were applied (BCP-ALL, T-ALL and AML, **Supplementary Table 1**). Sample acquisition was conducted using BD FACSCanto II or BD FACSLyric cytometers. Analysis of flow cytometry data was performed using Infinicyt 2.0 software.

### 2.3 Isolation, expansion and immunophenotyping of BM mesenchymal stromal cells

Mononuclear cells (2-4 x 10^6^) from BM were placed in culture with low glucose Dulbecco´s modified Eagle´s medium (DMEM, Gibco) supplemented with 10% fetal bovine serum (FBS, Gibco) and 100 U/ml of penicillin/streptomycin (Gibco) (17). MSC were then isolated by their plastic adherence properties according to the International Society for Cellular Therapy (18). Upon confluence, cell monolayers were trypsinized and reseeded for their expansion and biological characterization. All experiments were conducted with harvested cells from the second passage. MSC staining for flow cytometry analyses was performed according to our previous report (17) for surface molecules of interest, including CD90, CD73, CD105 and CD45 markers for general MSC identification, and CXCL12, CXCL11, Galectin-9 (LGALS9), CD39 and IDO1 for immune regulatory functions.

### 2.4 High dimensional reduction analysis

Age, frequencies of residual and leukemic hematopoietic cell populations and blast expression based on mean fluorescence intensity of ALOT were subjected to unsupervised clustering using the graph-based visualization method Uniform Manifold Approximation and Projection (UMAP) and Principal Component Analysis (PCA). The analysis was performed in Rstudio with the library “umap” and GraphPad Prism 10.0.3 (La Jolla, California USA), respectively.

### 2.5 Statistical analysis

Bar graphs show mean values and standard deviation (SD). GraphPad Prism version 10.0.2 for Windows (La Jolla, California USA) was used for data analysis. Differences within groups were established by the non–parametric Kruskal–Wallis with Dunn’s post–test to compare continuous variables. Relative risk (RR) and 95% confidence interval (CI) was calculated with Koopman asymptotic score. Relative risk values of detectable MRD (RR_MRDd_) were calculated by classification (each group *vs* PreB-ALL), sex (males *vs* females), age group I (1-9 *vs* 10-18 years old group), age group II (each age group *vs* 5-9 years old group), phenotype classification and age group (each group *vs* the ProB-PreB-ALL 1-9 years old group) and by UMAP cluster (each group *vs* group 1). To generate phenotypic signatures using Principal Component Analyses (PCA), we used frequencies and mean fluorescence intensities of each tumoral and immunological populations. Pearson’s test was used to determine correlations between signatures. p values < 0.05 were considered statistically significant.

## 3 Results

### 3.1 ProB-ALL with high expression of myeloid markers is prevalent in Mexican children from vulnerable regions

**Figure 1:**
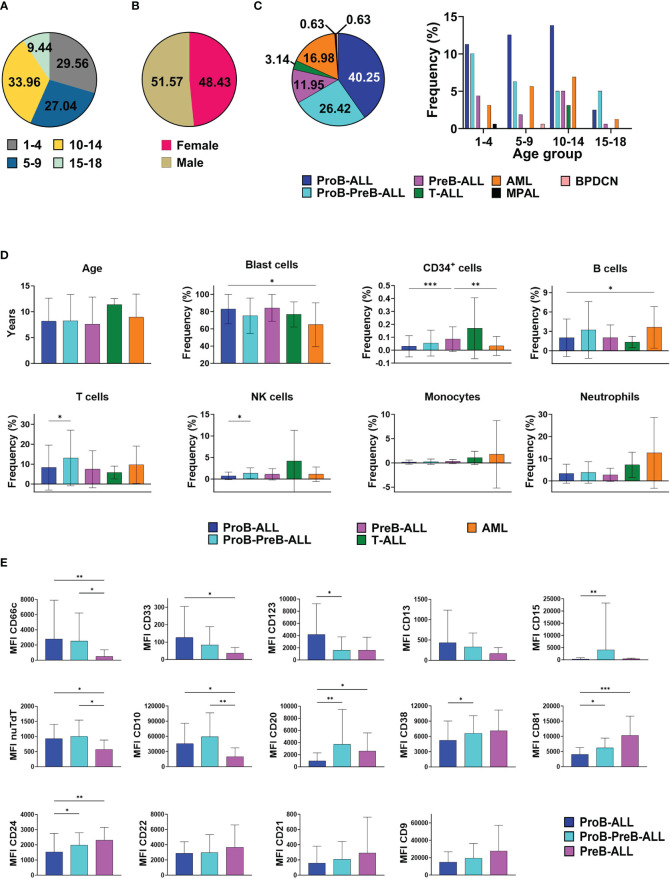
Cellular epidemiology of acute leukemias (AL) in Mexican children from Puebla, Oaxaca, and Tlaxcala. Distribution of AL cases by **(A)** age groups, **(B)** sex, **(C)** AL subtype according to age **(D)** Cell frequencies of normal and malignant hematopoietic populations at diagnosis by AL subtype. Bar graphs exclude MPAL and BPDCN due to the limited occurrence of cases (one each). **(E)** Marker expression patterns in B-ALL subtypes. AL, Acute Leukemia ALL, Acute Lymphoblastic Leukemia; AML, Acute Myeloid Leukemia; MPAL, Mixed Phenotype Acute Leukemia; BPDCN, Blastic Plasmacytoid Dendritic Cell Neoplasm; NK, Natural Killer; MFI, Mean Fluorescence Intensity; nuTdT, nuclear Terminal deoxynucleotidyl Transferase. * *p*<0.05, ** *p*<0.01, ***p<0.001. Bar plots shown mean ± standard deviation. n AL cases=159.

This study includes a cohort of 159 pediatric patients aged 1 to 17 years, with a median age at presentation of 9 years. The age distribution of cases shows the highest incidence occurring within the 10-14 age group, accounting for 33.96% of cases. While the age group of 1-4 years constituted 29.56% of cases and the 5-9 years age group accounted for 27.04%, adolescents 15-18 years old showed a notably lower incidence rate of 9.44% (**Figure 1A**). Among these patients, 51.57% were males, although females were more prevalent in the 1-4 age group, while males in the 10-14 age group (**Figure 1B**). At clinical diagnosis, the distribution of leukemia subtypes was: 78.62% of patients were classified as B-ALL (mainly categorized as ProB-ALL), 3.14% T ALL, 16.98% AML, 0.63% MPAL (mixed phenotype acute leukemia) and 0.63% blastic plasmacytoid dendritic cell neoplasm (BPDCN). A noticeable trend emerges, showing a positive correlation between the incidence of ProB-ALL cases and age, in contrast to the negative correlation between ProB-PreB cases and age. Remarkably, all cases of T-ALL were exclusively observed in the 10-14 age group (**Figure 1C**).

At the onset of AL, normal CD34^+^ hematopoietic stem and progenitor cells (HSPCs) coexists with malignant blasts within the same hematopoietic niche, so they and their progeny recurrently show a crucial reduction in cell frequencies. When investigating both residual and malignant cell populations to evaluate the degree of differentiation imbalance based on the AL subtype, we observed that patients with AML debut with a significantly lower tumor burden compared to those with ProB-ALL. Accordingly, ProB-ALL cases exhibit the lowest frequencies of normal CD34^+^ seminal cells, as well as T, B and NK lymphoid lineage cells (**Figure 1D**). Strikingly, ProB-ALL leukemic cells show higher expression levels of myeloid markers such as CD66c, CD33 and the immaturity marker CD123, along with the apparent downregulation of CD20, CD38, CD81 and CD24 expression. In contrast, PreB-ALL blasts display significantly lower expression levels of such infidelity markers, including CD66c and CD33, while the highest expression of CD81 and CD24 was recorded in this ALL subtype. Interestingly, the potentially transitional ProB-PreB-ALL subtype exhibited the highest co-expression of myeloid marker CD15 and lymphoid proteins such as nuTdT, CD10 and CD20 (**Figure 1E**).

### 3.2 Adolescents aged 10-18 years suffering ProB-ALL or AML face the highest relative risk of residual disease

To identify potential patient groups with more susceptibility to adverse events, we calculated the relative risk (RR) of detecting MRD based on variables such as AL subtype, sex, age and combined stratification of AL subtype and age range. Regarding AL subtype, patients with AML demonstrated a significantly higher risk. A tendency for higher risk was observed in patients with ProB-ALL, followed by patients with ProB-PreB-ALL, although these did not reach significance. No significant difference was observed between females and males. On the other hand, patients older than 10 years were found to have a 2.649-fold increased risk of detectable MRD. Of note, within this age range, patients aged 10-14 years exhibited the highest RR value. By combining AL subtypes and age, patients aged 10-18 with AML display a 10-fold increase in risk, followed by patients with ProB-ALL aged 10-18, with 7-fold increased risk (**Table 1**).

**Table 1:** Relative risk (RR) of detectable MRD by immunophenotype, sex and age in 159 pediatric acute leukemia cases.

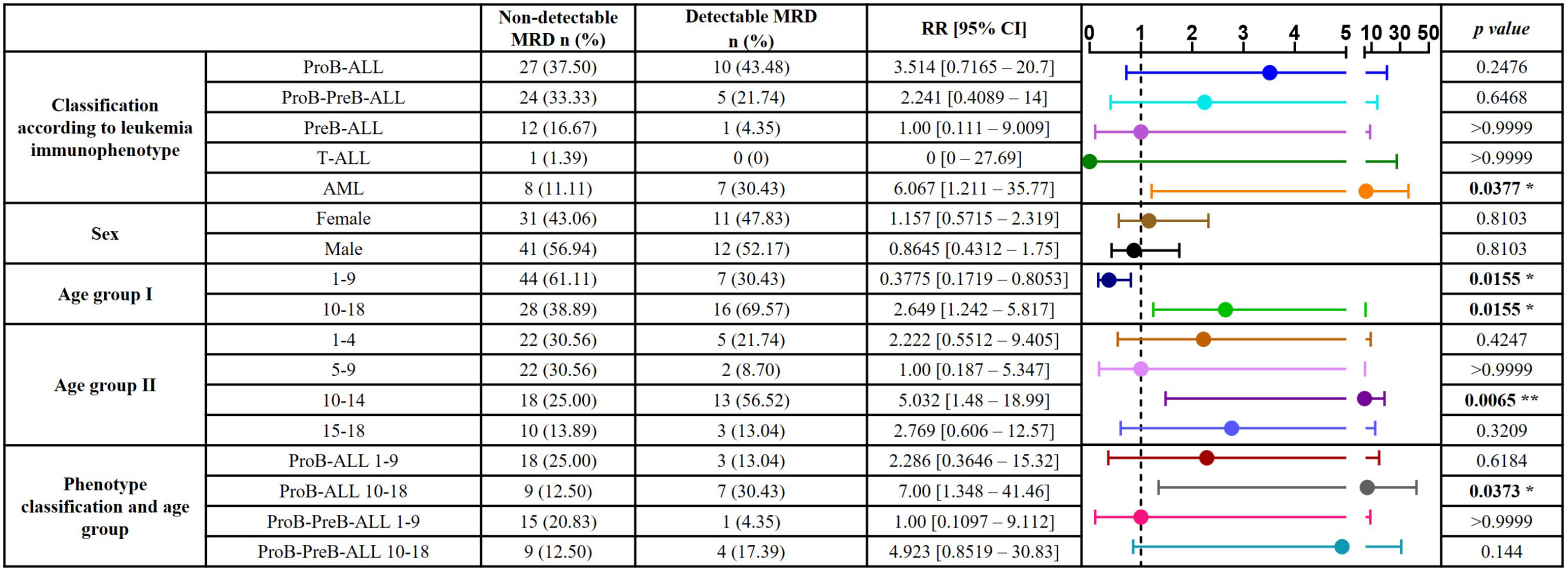

RR values were calculated by immunophenotype classification (each group vs PreB-ALL), sex (males vs females), age group I (1-9 vs 10-18 years old group), age group II (each age group vs 5-9 years old group), and by classification in each age group (each group vs the ProB-PreB-ALL 1-9 years old group). ALL, Acute Lymphoblastic Leukemia; AML, Acute Myeloid Leukemia; MRD, Measurable Residual Disease. * p<0.05, ** p<0.01. RR plot shown RR value ± 95% confidence intervals. Bold values means statistically significant.

### 3.3 UMAP-based clustering identifies a poor prognosis ProB-ALL subgroup

**Figure 2:**
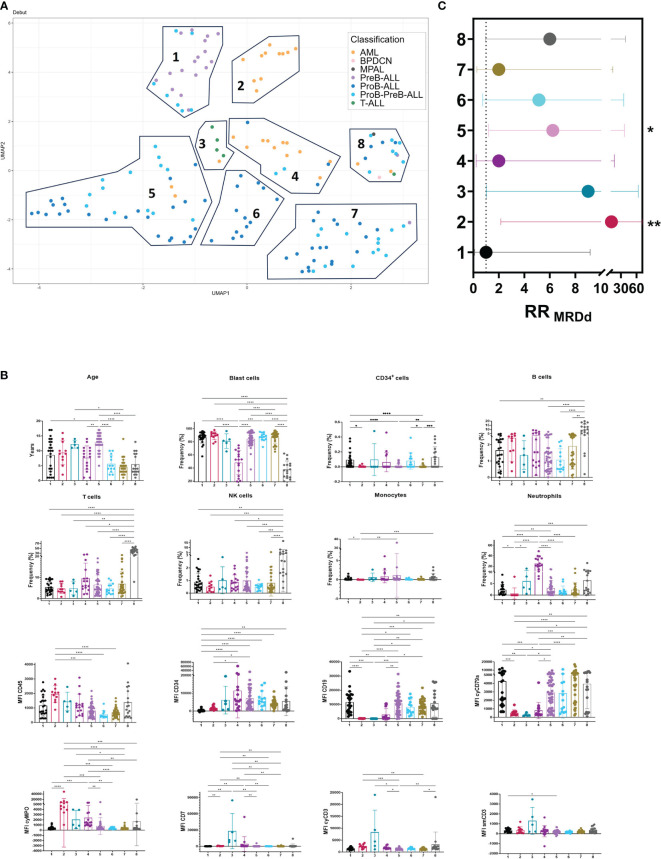
UMAP: an innovative approach to leukemic profiles of prognostic value in childhood acute leukemias. Risk stratification in acute leukemias was explored by Uniform Manifold Approximation and Projection (UMAP). **(A)** Identification of 8 leukemia clusters by UMAP analysis of 159 AL cases based on age, cell frequencies of normal and leukemic hematopoietic cell populations and expression levels of ALOT markers based on mean fluorescence intensity. **(B)** Cell populations and blast expression patterns in UMAP clusters. **(C)** RR values of detectable MRD (RR_MRDd_) were calculated by UMAP clusters (each group *vs* group 1). UMAP, Uniform Manifold Approximation and Projection; NK, Natural Killer; MFI, Mean Fluorescence Intensity; nuTdT, nuclear Terminal deoxynucleotidyl Transferase; cy-, cytoplasmic; sm-, surface membrane; RR, Relative risk; MRD, Measurable Residual Disease; MRDd, detectable MRD. * *p*<0.05, ** *p*<0.01, ***p<0.001, ****p<0.0001. Bar plots shown mean ± standard deviation. RR plot shown RR value ± 95% confidence intervals.

To identify patients at risk of developing adverse events, we used the high dimensional reduction algorithm UMAP, which generate unsupervised clusters based on factors such as age, the protein expression of leukemic cells (CD45, CD34, CD19, cyCD79a, cyCD3, smCD3, CD7 and cyMPO) and the frequencies of residual and pathological hematopoietic populations detected in the ALOT assay at the time of diagnosis. Our high precision analysis revealed eight distinct clusters (**Figure 2A**). Cluster 1 primarily comprises PreB-ALL cases, with only one exception, and includes some ProB-PreB cases. Of note, this cluster exhibited the lowest number of detectable MRD and was therefore used as the reference group for comparisons. Interestingly, cluster 1 had a significantly lower mean age than cluster 5, along with a higher frequency of normal CD34^+^ cells (HSPCs) and the lowest expression of CD34 marker on blast cells. AML cases were primarily clustered in groups 2 and 4. Although no significant difference in age was observed between them, group 2 exhibited the top risk of detectable MRD. This group was characterized by high frequency of blasts and higher expression levels of cyMPO, along with a reduction in the neutrophil and T cell compartments. In this group, leukemic cells lack CD34.

Group 3 mainly contained T ALL cases and one AML case with aberrant expression of CD7 and CD56 according to the AML panel, and a RR value of 9.00, although with no statistical significance. While groups 5 and 7 primarily consisted of ProB and ProB-PreB cases, group 5 had a higher risk, with a 6.231-fold increase compared to the 2-fold increase in group 7. Notably, group 5 included older patients with leukemic blast cells showing a tendency toward lower expression of cyCD79a. Group 6 had a RR value of 5.143, although it did not reach statistical significance. This group consists solely of ProB-ALL cases with mean age of 5.61, a low frequency of B cells and the lowest expression of CD45, this group warrants attention as, based on the classification of AL subtype and age, it is categorized with lower risk. Lastly, group 8 encompassed all 5 subtypes of acute leukemia. Interestingly, this group coincided with the lowest frequency of blasts, concomitant with the highest frequency of B, T and NK cells (**Figures 2B, C**).

### 3.4 PCA-based profiles define signatures between B-ALL clusters

**Figure 3:**
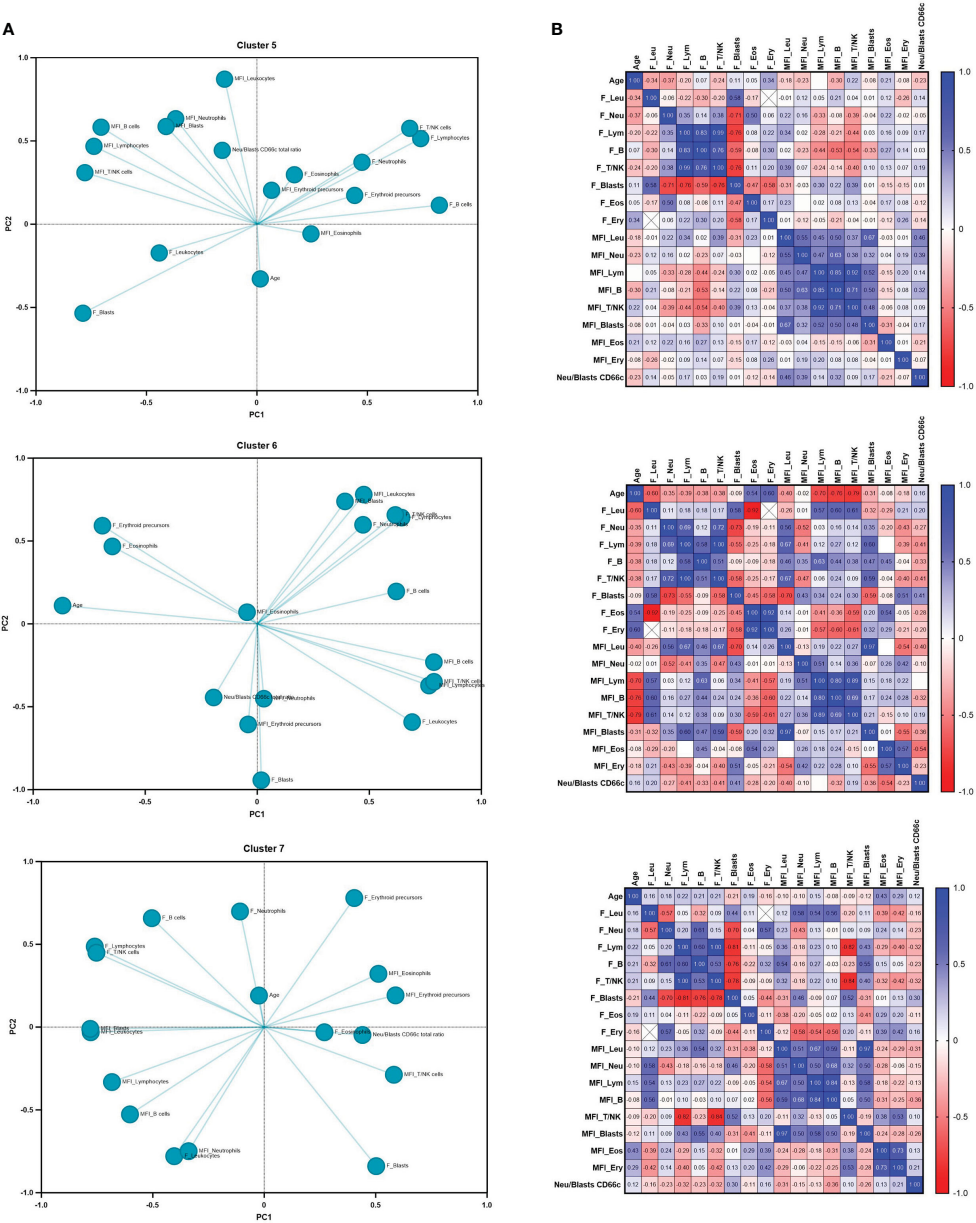
Unsupervised sub-stratification of BCP-ALL defines unique phenotypic profiles. **(A)** Principal component analysis (PCA) and **(B)** Pearson correlation matrix of 5, 6 and 7 groups by UMAP clusters were performed based on age, cell frequencies and mean fluorescence intensity of normal (leukocytes, neutrophils, lymphocytes, B cells, T/NK cells eosinophils and erythroid precursor cells) and leukemic hematopoietic cell populations. The ratio of CD66c expression on neutrophils and blasts was used as indicator of level expression of this aberrant marker. *p* values are shown in correlation matrix.

Although it is true that the evaluation of unique markers provides invaluable information about the behavior of the tumor, the understanding of its interaction with the surrounding microenvironment must be evaluated considering each of the variables that identify the complex intrinsic and extrinsic relationships of tumor cells. For this, an unsupervised analysis of all variables was performed. Principal component analysis was based on age, cell frequencies, and mean fluorescence intensity of normal hematopoietic (leukocytes, neutrophils, lymphocytes, B cells, T/NK cells, eosinophils, and erythroid precursor cells) and leukemic cell populations to identify unique profiles within groups 5, 6, and 7 (**Figures 3A, B**). The proportion of CD66c expression in neutrophils and blasts was used as an indicator of the level of expression of this aberrant marker. Loadings representing the correlation between the original variables and the principal components are shown (**Figure 3A**). The loadings of a PCA indicate the contribution of each variable to the corresponding principal component. Variables with high loadings (positive or negative) have a significant impact on that component, therefore, the loading profile between each signature is different. This highlighting that although groups 5 and 7 grouped by the UMAP are composed of ProB and ProB-PreB subtype leukemias, the identity profile shown by PCA is different between these groups. Leukocytes and neutrophils cell frequencies, MFI from blasts, eosinophils, erythroid precursors, and the expression ratio of the CD66c marker, clearly generate particularly different profiles between these clusters.

### 3.5 A subtype of ProB- and ProB-PreB-ALL, but not PreB-ALL, are supported by a suppressive niche

**Figure 4:**
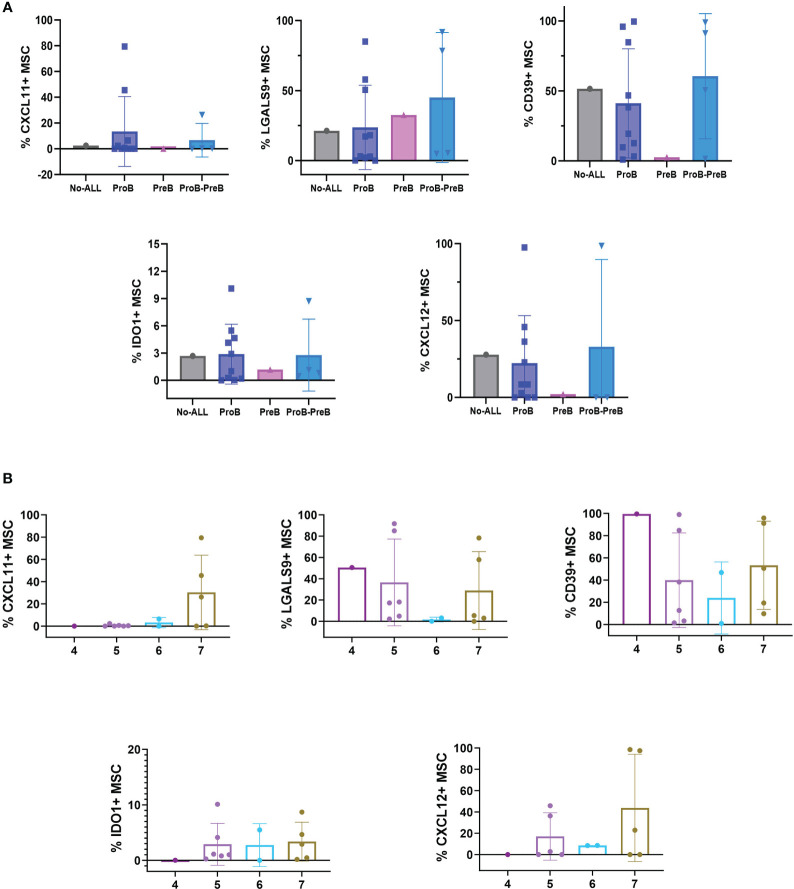
B-ALL BM niches are distinct in leukemic groups at debut. **(A)** Phenotypic evaluation of MSC stratified by B-ALL subtypes and No ALL-MSC. No ALL = 1, ProB-ALL = 10, PreB-ALL = 1, ProB-PreB-ALL = 4. **(B)** Percentage of CXCL11-, LGALS9-, CD39-, IDO1- and CXCL12- expressing MSC on clusters 4 (n=1), 5 (n=6), 6 (n=2) and 7 (n=5) according UMAP subclassification for Pro-B-ALL and Pro-B-Pre-B-ALL subtypes. B-ALL, B-cell precursor acute lymphoblastic leukemia; MSC, mesenchymal stromal cells; CXCL, chemokine (C-X-C motif) ligand (CXCL); LGALS9, galectin 9; IDO, Indoleamine 2,3-dioxygenase. Bar plots shown mean ± standard deviation.

Here, we investigated the expression level of a number of mesenchymal markers to identify a potential microenvironmental signature by the unique ProB-ALL subtype. Interestingly, our results suggest phenotypical differences between B-ALL subtypes. Exhaustive flow cytometry of CD105^+^ CD73^+^ CD90^+^ and CD45^-^ MSCs cells, a likely suppressive phenotype, characterized by expression of CXCL11, Galectin-9 (LGALS9), CD39 and indolamine 2,3-dioxygenase (IDO1), was observed in ProB and ProB-PreB MSCs compared to PreB MSCs (**Figure 4A**). Lower expression of CXCL12 by BCP-ALL MSCs than No-ALL was confirmed (17).

To determine whether the phenotypic characterization of MSCs could also be associated with any of the clusters depicted from UMAP analysis, we identified the leukemia cases corresponding to the mesenchymal subtypes within the UMAP clusters (**Figure 4B**). While CXCL11 was apparently a marker for cluster 7, LGALS9 and CD39 show the lowest expression in cluster 6. In contrast, clusters 5 and 7 exhibit high percentages of MSCs positive for these markers, suggesting they may promote suppressive conditions in some ProB and ProB-PreB-ALL. Preliminary, cluster 4 lacks expression of IDO while showing the highest cell frequencies of CD39 positive MSCs.

### 3.6 A risk profile for children over 10 years of age emerges from integration of the leukemic phenotype, the immunological context, and the tumor microenvironment

We focused on a subset of B-ALL cases, the ProB subtype cases within the UMAP clusters 5-7. Our primary goal was to gain insights into the immune context, tumor immunophenotype and microenvironment components in order to identify a potential risk-prognostic profile.

**Figure 5:**
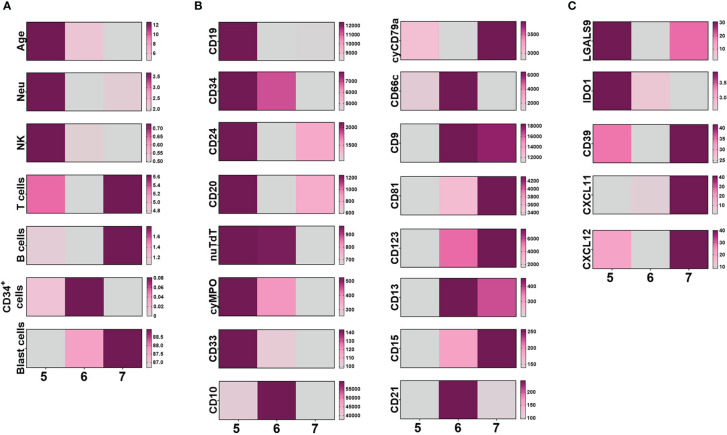
Identity of ProB-ALL within UMAP clusters. The comparison of ProB-ALL immunological context **(A)**, malignant cell immunophenotype **(B)** and mesenchymal stromal microenvironment immunophenotype **(C)** within UMAP clusters 5 to 7 is represented by heatmaps. Mean values of age, frequency of hematopoietic cell populations, MFI of tumor cell markers and percentage of mesenchymal stromal cells expressing CD markers, are shown. UMAP, Uniform Manifold Approximation and Projection; NK, Natural Killer; MFI, Mean Fluorescence Intensity; nuTdT, nuclear Terminal deoxynucleotidyl Transferase; MPO, myeloperoxidase; CXCL, C-X-C motif chemokine; IDO1, Indoleamine-pyrrole 2,3-dioxygenase; cy-, cytoplasmic; sm-, surface membrane; CD, cluster differentiation.

Cluster 5, characterized by the highest rate of MRD, predominantly consisted of patients older than 10 years, showing a tendency for increased neutrophils and NK cell frequencies and less burden of blast cells, compared to values from clusters 6 and 7 (**Figure 5A**). HSPC normal counterpart is exceptionally low in ProB-ALL from cluster 5. Strikingly, when analyzing the immunophenotype of tumor cells within this cluster, an upward trend in the expression levels of CD19, CD34, CD24, CD20, nuTdT, cyMPO, and CD33 was recorded (**Figure 5B**). Conversely, there was a downward trend in the expression of CD9, CD81, CD123, CD13, CD15, and CD21 markers. Regarding the microenvironment profile, cases from cluster 5 displayed a tendency toward elevated expression of Gal9 and IDO, along with reduced expression of CXCL11 in their MSC, compared to their counterparts from clusters 6 and 7 (**Figure 5C**). Thus, a different tumor ecosystem in children who show a higher risk of relapse is suggested.

## 4 Discussion

Due to the high incidence of relapse in Mexican children from vulnerable populations, there is an urgent need of a comprehensive prognostic profile in those who debut even with a standard risk diagnosis to precisely target the risk and ultimately improve their clinical outcomes.

In acute leukemias, the principal contribution to disease free survival is provided by an adequate treatment of the disease, according to patient risk stratification and identifying relapse factors (6). Although the wide range of immunophenotypic and molecular profiles of leukemic cells contribute to the risk of relapse (19), the identification of a single biomarker associated with prognosis has proven to be insufficient. Of note, leukemic burden, and progression in acute leukemias are shaped not only by the neoplastic cell component but are also influenced by elements within the tumor microenvironment (20).

Here, we conducted for the first time, an integral analysis of the immunophenotypic profile, hematopoietic and microenvironmental cell populations, and age distribution among 159 pediatric patients recruited from five hospitals located in Puebla, Oaxaca and Tlaxcala, Mexico, from March 2022 to June 2023. The highest incidence was registered within the 10-14 years age group, with a male-to-female ratio of 0.74 among children aged 1 – 4 years and 1.35 among those aged 10-14 years. This differs from the findings of the Cancer Registry in Children and Adolescents of Mexico, where the highest incidence is recorded among children aged 1–4 years, and a predominance of male patients is observed across all age groups (21). A total of 35.22% of the cohort had a high-risk diagnosis due to the age group (>10 years old). It is worth noting that the absence of clinical parameters, such as leukocyte count and molecular biology test, may potentially lead to an underrepresentation of the number of cases within this specific category.

ALL emerged as the most prevalent lineage affected, accounting for the substantial 81.76% of all cases. Within the ALL category, the B-cell subtype was predominant at 96.15%, while the T-cell subtype was observed in 3.85% of cases. This, again, contrasts with the worldwide distribution of ALL, which consists of approximately 85% B-cell subtype and 15% T-cell subtype cases (22). Among the B ALL cases 84.8% displayed CD34 expression, which aligns with previous work indicating that CD34 is present on the outer membrane of blast cells in 60-83% of patients with B-lineage ALL (23).

At the debut of the disease, leukemic cells exert a detrimental impact on both resident HSPC and the microenvironment (24). Our findings revealed that, on average, blast cells occupied 77% of the bone marrow space. This displacement led to a reduction in the frequency and functionality of the normal progenitor compartment. Here, the ProB-ALL cases exhibited lower frequencies of normal CD34^+^ cells, along with reduced frequencies of B, T and NK cells, as previously reported by Ramírez et al. (25). Accordingly, Balandrán et al. reported that lower cell frequencies of HSPC in ProB-ALL correlated to high-risk prognosis at disease debut (10). Demanou-Peylin et al. reported that, in the context of B-ALL at the initial diagnosis, the limited number of HSPCs demonstrated diminished hematopoietic potential, with a high mortality rate, which can be explained by their low intrinsic functional activity (24). In AL, consequently, this results in severe pancytopenia that clinically includes anemia, recurrent infections and petechiae (1).

Aberrant immunophenotypes have been investigated as prognostic factors in AL by several studies (26–28). In this cohort, we evaluated the presence of cross-lineage myeloid markers such as CD66c, CD33, CD13 and CD15, some associated with specific molecular abnormalities including NG2 and CD9, and overexpression of B-cell lineage markers in B-ALL cases. Notably, our findings reveal that the ProB-ALL subtype display significantly elevated expression levels of myeloid markers, including CD66c, CD33 and CD13, concomitant by a tendency toward lower expression of CD9, this profile contrasts to the PreB-ALL cases. The glycoprotein CD66c is abnormally expressed on blast cells, particularly in cases with the t(9;22) translocation that originates the *BCR::ABL1* fusion protein and in some hyperdiploid molecular subgroups (26). *BCR::ABL* positive cases often display increased expression of myeloid markers like CD13 and CD33, alongside a CD34^hi^ and CD38^lo^ profile, typically without expression of CD117 (29). Regrettably, among our B-ALL cases, approximately 44% correspond to the ProB subtype. The identification of this B-ALL subtype with this profile increases the likelihood of encountering this gene fusion, which is associated with a poor prognosis. The ProB-PreB-ALL cases exhibited the highest expression of CD15, CD10 and CD20. Positive CD10 expression has been associated with favorable clinical outcomes in children (30). Indeed, Kulis et al., found a strong association between the high expression of CD10 and the presence of the *ETV6::RUNX1* gene fusion (9, 31). The analysis by Bhojwan et al. revealed that *ETV6::RUNX1* is associated with patients aged 1-9 years, categorized as low-risk before treatment, and displaying lower levels of MRD on day 19 of therapy (p<0.001) (32). Very interestingly, in the Mexican population, the occurrence of *ETV6::RUNX1*, which is generally associated with a standard prognosis, is less common. Instead, rearrangements involving *CRLF2* and *iAMP21*, which confer a high risk for leukemia, are more frequently observed among Mexican patients (8). The prognostic significance of CD20 in pediatric B-ALL has yielded conflicting results across studies, in contrast to adult B-ALL that have consistently shown that CD20 positivity is typically linked to a less favorable outcome (26).

Based on the computed relative risk (RR) values for detectable MRD reported in this study, it was evident that patients with AML exhibited the highest risk of detecting residual disease. Among all cases of B-ALL, the ProB-ALL subtype showed a notable tendency towards the highest RR value, followed by ProB-PreB-ALL. These subcategories are primarily distinguished by the expression of CD34, a transmembrane protein initially identified on HSPCs. CD34 plays a pivotal role in facilitating the attachment of these progenitor cells to the stromal microenvironment components, thereby supporting their growth and differentiation (33). Our observations align with the findings of Modving et al., who demonstrated that a lack of CD34 expressions serves as a favorable prognostic factor in ALL (34). Conversely, high CD34 expression is associated with poor therapy response and an altered gene expression profile resembling that of migrating cancer stem-like cells (35). Our findings revealed that the age group between 10 and 14 years exhibited a significant RR for detectable MRD. This contrasts with the previously reported data indicating that individuals aged 15 to 19 experience the highest levels of aggressiveness and mortality rates (4). Among the cases of B-ALL recorded in patients aged 10 years and older, it was observed that the ProB-ALL subtype exhibited a substantial increase in the likelihood of presenting adverse events. In contrast, the PreB-ALL subtype continued to demonstrate a favorable prognosis even within this age group.

UMAP analyses have clearly shown that the highest RR value is in a cluster of AML cases, where blast cells exhibited the absence of CD34 expression and a trend of higher cyMPO expression. The literature offers no definitive consensus regarding the prognostic significance of CD34 expression in AML. Some studies have reported that its expression is associated with a favorable prognosis (33), while others have suggested that CD34 expression correlates with poor clinical outcome, and CD38-negative CD34-positive leukemic cells demonstrate enhanced leukemia-initiating capacity and exhibit stem-like features, including a quiescent phenotype and increased expression of adhesion-related molecules such as CD44, CXCR4, integrins, as well as the growth guidance receptor ROBO4 (35). Significantly, the UMAP analysis identified an immunophenotype profile associated with a poor prognosis in AML cases, despite the absence of statistical differences in age between clusters 2 and 4. To investigate deeper into ProB-ALL cases within cluster 5, which predominantly comprised adolescent patients older than 10 years old, our risk stratification analysis, exhibited a distinct tumor cell profile in cluster 5, compared to ProB-ALL cases within cluster 7, which had a lower RR_MRDd_. Firstly, there was a higher expression of CD34, a marker often linked to poor outcomes in ALL. Secondly, the elevated expression of CD20, typically associated with a less favorable prognosis in adult patients. Additionally, cluster 5 exhibited significantly higher levels of cyMPO (higher expression than other B-ALL cases but lower compared to AML or MPAL cases, that cannot be categorized as positive), a marker known to be associated with an increased risk of relapse and even worse event-free survival, even in the absence of other myeloid markers (36). Also, the presence of the aberrant marker CD33 (**Figure 5**). Notably, the ProB-ALL cases in cluster 5 displayed several features associated with a less favorable prognosis.

Novel immunological treatments are gaining interest in the treatment of ALL, so it is elemental to examine ALL immunobiology in more detail (37). The tumor microenvironment (TME) plays a critical role in cancer development (from the first steps of initiation, through invasion and metastasis) (34, 38). Within this intricate milieu, it is becoming increasingly evident that understanding the dynamic interplay between tumor cells and their microenvironment is of greatest importance. The analyses of TME may also provide more detailed information on tumor ecosystems and predict the response and applicability of immunotherapy (39). Accordingly, at least 2 differential niches of MSCs that may have clinical implications for ALL patients, and a detailed transcriptional fingerprint in healthy BM samples have enabled to define phenotypically and functionally distinct stromal subsets (40).

Inflammation is an important hallmark of cancer and is associated with many types of malignancies (34). The adaptability of the BM niche to stress suggests that there may be premalignant niches which could support the expansion of clones with some growing advantages. BM-MSC can play both inflammatory and anti-inflammatory functions. MSC derived from a healthy donor can suppress T-cell proliferation and NK cytotoxicity by the expression of PD-L1, IL-10, IDO1 and TGFβ (13, 41). Thus, the functional role of MSCs depends on the components within the microenvironment (42).

Furthermore, adenosine production increases during inflammation by CD39 and CD73 that act sequentially limiting immune response. CD39 is an ecto-nucleoside enzyme that binds ATP and converts it to adenosine. By converting ATP into AMP, CD39 increases adenosine production via CD73, which is also an ecto-enzyme that acts by hydrolyzing AMP into adenosine. The co-expression of both ectoenzymes is high in human tumors (41, 43). The BM niches recruit Treg cells via CD39 activity (13). Although CD73 is a major cell surface marker for MSCs, the knowledge about the role of this molecule in the regulation of these cells is very low (44). CD73 apparently participates in tumor immune-escape by inhibiting activation, clonal expansion, and homing of tumor-specific T cells, impairing tumor cell killing and enhancing the conversion of antitumor type 1 macrophages into protumor type 2 macrophages. Thus, CD73 expressed on stromal cells or tumor cells contributes to tumor-induced immune suppression (44). On the other hand, CXCL11 is a chemokine with a key role in immune and inflammatory responses by promoting the recruitment and activation of different subpopulations of leukocytes. IL1β, IFN-γ and IFN-β have been reported to increase the production of CXCL11 (45). This chemokine has the highest affinity with CXCR3, and also can bind CXCR7 (46). Our discovery of its absence in the niches supporting the ProB subtype, associated with a higher risk of relapse in older children, aligns with the concept of an immunosuppressive identity present from the early stages of the disease.

This study has notable strengths, such as the inclusion of samples collected at the time of diagnosis from vulnerable regions across the country, which is particularly significant as most of the existing literature consists primarily of patient cohorts in Mexico City. By encompassing a more geographically diverse patient population, our study allows for a comprehensive analysis of the interplay between tumor and microenvironment components and the age-specific characteristics of this high-mortality region. Limitations of our study include the absence of detailed information on key laboratory parameters, including leukocyte, neutrophil, and platelet counts. Additionally, our work lacks comprehensive data on comorbidities and the occurrence of adverse events, such as opportunistic infections, which can significantly impact patient outcomes. Furthermore, the limited availability of monitorization test (MRD) from only 95 patients in the cohort (59.7%), may restrict the generality of our conclusions. Also, we have a small sample size of 5 patients with T-ALL, this limited number of cases may impact the observed trends, warranting careful consideration and potential cautious interpretation. A more extensive dataset would provide a more robust basis for obtaining comprehensive insights and making broader recommendations.

Here, we successfully identified immunophenotypic profiles associated with clinical prognosis, addressing an urgent need considering the constrained access to molecular biology tests within public health systems, which are used to assign AL cases to risk groups. These limitations arise due to the considerable expenses involved and a shortage of human resources. Overall, this study reveals the substantial importance of risk stratification beyond the tumor immunophenotype, especially in Mexican patients who present greater vulnerability conditions. We have discovered the integrated ecosystem of a leukemia with ProB tumor characteristics and myeloid elements, which grows at the expense of normal hematopoietic differentiation and in a potentially suppressive microenvironmental context. It is highly relevant to thoroughly investigate the signals and environmental factors that induce this phenotype for further intervention.

## Data availability statement

The raw data supporting the conclusions of this article will be made available by the authors, without undue reservation.

## Ethics statement

The studies involving humans were approved by Comisión Nacional de Investigación Científica of the Mexican Social Security Institute (R-2020-785-177). The studies were conducted in accordance with the local legislation and institutional requirements. Written informed consent for participation in this study was provided by the participants’ legal guardians/next of kin.

## Author contributions

RR-R: Conceptualization, Methodology, Writing – original draft, Writing – review & editing, Data curation, Formal analysis, Visualization. GZ-H: Conceptualization, Data curation, Formal analysis, Methodology, Visualization, Writing – original draft, Writing – review & editing. JL-B: Data curation, Writing – review & editing. LL-G: Methodology, Writing – review & editing. AR-C: Methodology, Writing – review & editing. LA-H: Writing – review & editing, Methodology. CT-P: Methodology, Writing – review & editing. DA-A: Methodology, Writing – review & editing. DC-A: Resources, Writing – review & editing. AV-O: Methodology, Writing – review & editing. JS-P: Resources, Writing – review & editing. LG-S: Resources, Writing – review & editing. VT-C: Resources, Writing – review & editing. NL-S: Resources, Writing – review & editing. MG-H: Writing – review & editing, Resources. LC-C: Resources, Writing – review & editing. KA: Methodology, Writing – review & editing. FD: Writing – review & editing. MdC-M: Writing – review & editing. GJ-A: Resources, Writing – review & editing. JB: Validation, Writing – review & editing. SP-T: Funding acquisition, Writing – review & editing. CF-G: Supervision, Writing – review & editing. PZ-R: Supervision, Writing – review & editing. EL-A: Writing – review & editing. AT-G: Funding acquisition, Writing – review & editing. CD-M: Writing – review & editing. LB: Writing – review & editing. JN-E: Formal analysis, Resources, Writing – review & editing. MC-G: Writing – review & editing. EÁ-B: Writing – review & editing. DR-R: Conceptualization, Writing – review & editing, Data curation, Formal analysis, Supervision, Visualization, Writing – original draft. RP: Funding acquisition, Writing – review & editing, Formal analysis, Project administration, Supervision, Writing – original draft, Conceptualization.
