# Supplementary material for: Subclassification of B-acute lymphoblastic leukemia according to age, immunophenotype and microenvironment, predicts MRD risk in Mexican children from vulnerable regions
Source: Front Oncol. 2024 Jan 5;13:1304662. doi: 10.3389/fonc.2023.1304662 (PMC10796993; doi:10.3389/fonc.2023.1304662)
Supplement: Supplementary file 1 [file Table_1.docx]

| **Supplementary Table 1** | | | | | | | | |
| --- | --- | --- | --- | --- | --- | --- | --- | --- |
|  | **V450** | **V500** | **FITC** | **PE** | **PerCP-Cyanine 5.5** | **PE-Cyanine 7** | **APC** | **APC-H7** |
| **Acute Leukemia Orientation Tube (ALOT)** | | | | | | | | |
| **Tube 1** | cyCD3 | CD45 | cyMPO | cyCD79a | CD34 | CD19 | CD7 | smCD3 |
| **BCP-ALL panel** | | | | | | | | |
| **Tube 1** | CD20 | CD45 | CD58 | CD66c | CD34 | CD19 | CD10 | CD38 |
| **Tube 2** | smIgκ | CD45 | cyIgμ | CD33 | CD34 | CD19 | smIgμ and CD117 | smIgλ |
| **Tube 3** | CD9 | CD45 | nuTdT | CD13 | CD34 | CD19 | CD22 | CD24 |
| **Tube 4** | CD21 | CD45 | CD15 | NG2 | CD34 | CD19 | CD123 | CD81 |
| **T-ALL panel** | | | | | | | | |
| **Tube 1** | cyCD3 | CD45 | nuTdT | CD99 | CD5 | CD10 | CD1a | smCD3 |
| **Tube 2** | cyCD3 | CD45 | CD2 | CD117 | CD4 | CD8 | CD7 | smCD3 |
| **AML panel** | | | | | | | | |
| **Tube 1** | HLA-DR | CD45 | CD16 | CD13 | CD34 | CD117 | CD11b | CD10 |
| **Tube 2** | HLA-DR | CD45 | CD35 | CD64 | CD34 | CD117 | CD300e | CD14 |
| **Tube 3** | HLA-DR | CD45 | CD36 | CD105 | CD34 | CD117 | CD33 | CD71 |
| **Tube 4** | HLA-DR | CD45 | nuTdT | CD56 | CD34 | CD117 | CD7 | CD19 |
| **Tube 5** | HLA-DR | CD45 | CD15 | NG2 | CD34 | CD117 | CD22 | CD38 |
| **Tube 6** | HLA-DR | CD45 | CD42a and CD61 | CD203c | CD34 | CD117 | CD123 | CD4 |
| **Tube 7** | HLA-DR | CD45 | CD41 | CD25 | CD34 | CD117 | CD42b | CD9 |

**Supplementary Table 1. Panels for immunophenotyping of acute leukemias according to Euroflow™ platform.** ALOT, Acute leukemia orientation tube**;** FITC, Fluorescein isothiocyanate; PE, Phycoerythrin; PerCP, Peridinin-chlorophyll-protein; APC, Allophycocyanin; MPO, Myeloperoxidase; TdT, Terminal deoxynucleotidyl transferase; NG2, neural/glial antigen 2; HLA-DR, Human leukocyte antigen –DR isotype; ALL, Acute lymphoblastic leukemia; AML, Acute myeloid leukemia.
